# Improved prediction of PARP inhibitor response and identification of synergizing agents through use of a novel gene expression signature generation algorithm

**DOI:** 10.1038/s41540-017-0011-6

**Published:** 2017-03-06

**Authors:** Daniel J. McGrail, Curtis Chun-Jen Lin, Jeannine Garnett, Qingxin Liu, Wei Mo, Hui Dai, Yiling Lu, Qinghua Yu, Zhenlin Ju, Jun Yin, Christopher P. Vellano, Bryan Hennessy, Gordon B. Mills, Shiaw-Yih Lin

**Affiliations:** 1 Department of Systems Biology, MD Anderson Cancer Center, Houston, TX 77030 USA; 2 Centre for Systems Medicine, Royal College of Surgeons in Ireland, 123 St. Stephen’s Green, Dublin 2, Ireland

## Abstract

Despite rapid advancement in generation of large-scale microarray gene expression datasets, robust multigene expression signatures that are capable of guiding the use of specific therapies have not been routinely implemented into clinical care. We have developed an iterative resampling analysis to predict sensitivity algorithm to generate gene expression sensitivity profiles that predict patient responses to specific therapies. The resultant signatures have a robust capacity to accurately predict drug sensitivity as well as the identification of synergistic combinations. Here, we apply this approach to predict response to PARP inhibitors, and show it can greatly outperforms current clinical biomarkers, including *BRCA1/2* mutation status, accurately identifying PARP inhibitor-sensitive cancer cell lines, primary patient-derived tumor cells, and patient-derived xenografts. These signatures were also capable of predicting patient response, as shown by applying a cisplatin sensitivity signature to ovarian cancer patients. We additionally demonstrate how these drug-sensitivity signatures can be applied to identify novel synergizing agents to improve drug efficacy. Tailoring therapeutic interventions to improve patient prognosis is of utmost importance, and our drug sensitivity prediction signatures may prove highly beneficial for patient management.

## Personalized medicine: Signature-guided cancer therapy

Personalized cancer therapy is one of the holy grails of oncology, as the ability to determine what treatment would best benefit a patient would serve not only to improve outcomes, but also mitigate side effects from less effective treatments. Here, we develop algorithms to predict what patients will respond to a given therapeutic modality, as well as ways to specifically target any observed phenotype, by integrating large scale data sets that profile cancer cell line gene expression and sensitivity to hundreds of drugs. Furthermore, we show how these gene expression signatures can be used to predict novel synergizing agents to further enhance the efficacy of these therapeutics. Taken together, this work stands to advance the era of personalized medicine by enabling precision medicine approaches in the clinic.

## Introduction

The personalized management of cancer relies on an exponential understanding of various cancer types, and of their subtypes, at both the genotypic and phenotypic levels.^1^ On a single molecule level, pre-clinical pharmaceutical testing in cancer cell line panels have guided early-stage clinical trials, such as with the use of gefitinib for epidermal growth factor receptor mutant lung cancers, imatinib mesylate for the fusion BCR-Abl oncogene in leukemia,^2^ and trastuzumab or lapatinib in HER2/ERBB2 amplified breast cancers.^3, 4^

Unfortunately, for the majority of therapeutic molecules, a single gene assay is insufficient to accurately predict drug response. Transcriptomic analysis represents one of the most promising approaches to overcome this challenge, and relies on robust gene expression signatures designed to capture the core common features indicative of drug sensitivity, regardless of their precise molecular origin.^5^ Rapid technological advances are quickly making clinical implementation of these multi-gene signatures feasible.^6^ For example, the 50-gene Prosigna based on the PAM50 gene set has been FDA approved^7^ and a 70-gene signature led to the development of MammaPrint, a commercially available DNA microarray that aids in the prediction of low-grade breast cancer prognosis, has recently completed phase III trials.^8–10^ Other signatures have shown promise to predict genomic instability in cancers.^11^ Many studies have suggested that human cancer cell lines model many “omic” aspects of tumors, thereby making them representative proxies for the identification and evaluation of effective therapeutic interventions.^12–16^ However, it has been challenging to implement successful approaches that leverage transcriptomic data from cells lines to predict patient responses. Here, we present a novel algorithm termed iterative resampling analysis to predict sensitivity (IRAPS) and demonstrate its utility on two agents that lack ideal markers for therapeutic response: cisplatin and poly (ADP-ribose) polymerase (PARP) inhibitors.

In contrast to the above-mentioned targeted therapies that rely on mutations/amplifications in a single gene for identification of patients likely to benefit, PARP inhibitors work more indirectly by synthetic lethality in patients with mutated *BRCA1* or *BRCA2*.^17, 18^ Both *BRCA1* and *BRCA2* are key components of the homologous recombination (HR) double-stranded break DNA repair pathway, resulting in increased risk of developing breast, ovarian, lung, bladder, and other cancers if they are mutated. It is proposed that PARP inhibition (PARPi) selectively targets BRCA-mutant cells by increasing DNA single-stranded breaks that result in irreparable DNA double-strand breaks during replication, culminating in cell death. Early trials in treating BRCA-mutant ovarian cancer patients were so successful that olaparib from AstraZeneca was granted accelerated approval for BRCA-mutant ovarian cancer and rucabarib from Clovis Oncology was given breakthrough therapy designation by the FDA.^19^

Despite this promise, stratification of patients for PARP inhibitor therapy by BRCA status is proving suboptimal, with the majority of BRCA-mutant patients failing to show objective responses,^19^ clearly necessitating approaches for better identification of patient populations for PARPi treatment. Here, we demonstrate the power of our novel IRAPS algorithm by developing gene expression signatures to predict response to PARPi as well as to the chemotherapy agent cisplatin. This algorithm integrates gene expression data from hundreds of solid tumor cell lines with known sensitivity to pharmaceutical agents. This broad panel of cell lines was randomly sampled over 1000 iterations to determine differentially expressed genes, before final optimization of the signature on the desired cancer type. In addition to demonstrating high accuracy in cell line panels, these signatures were capable of predicting patient survival following cisplatin treatment and response of both primary patient-derived tumor cells (PTDCs) and patient-derived xenografts to PARPi. We also show how these signatures can be used to identify novel synergizing agents to improve therapeutic efficacy. Finally, we demonstrate how documented gene expression signatures can be leveraged to identify therapeutic targets for previously known molecular phenotypes such as BRCAness. Taken together, this work provides a method for development of robust actionable gene expression signatures capable of improving clinical patient outcomes.

## Results

### IRAPS of cancer cell lines to therapeutic agents

**Fig. 1:**
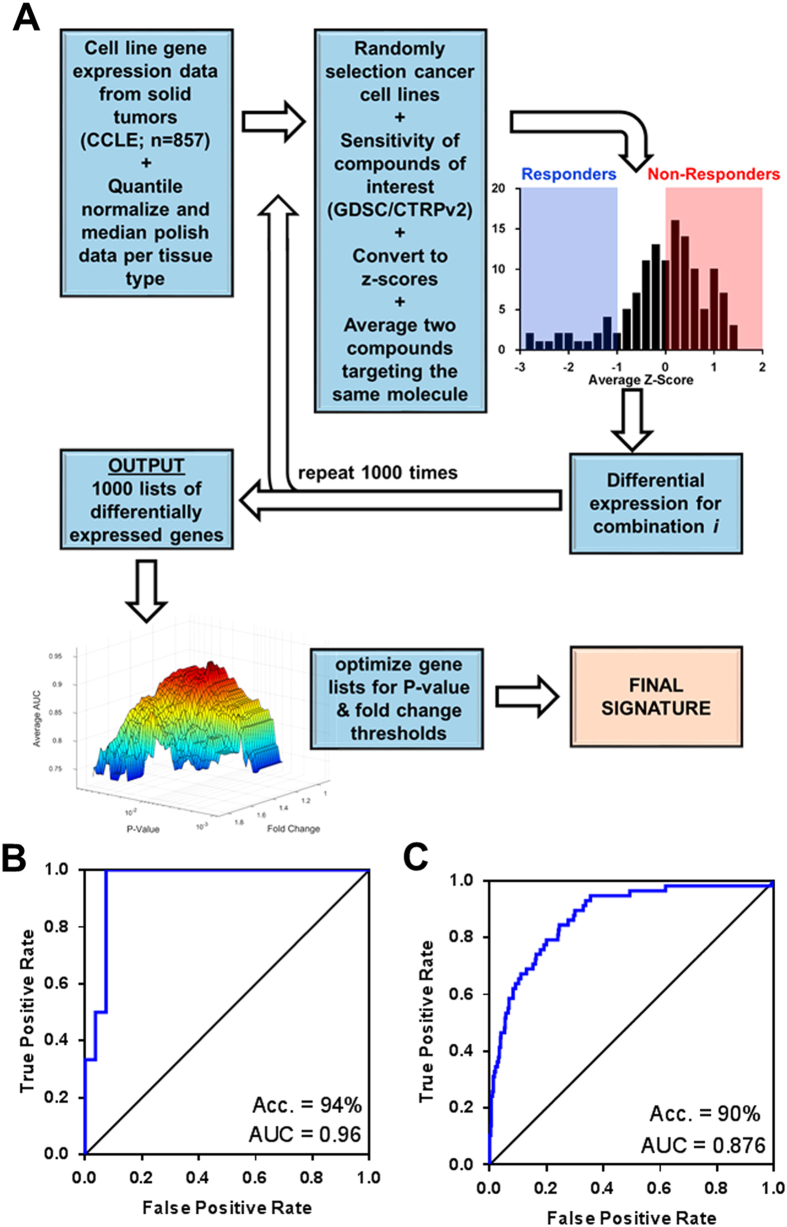
Overview of IRAPS **a** 857 solid-tumor cancer cells were stochastically and iteratively sampled for pharmaceutical agent sensitivity or insensitivity determination. Differential expression for the combination of two therapeutic agents per target was calculated. This process was repeated 1000 times for the generation of 1000 different lists of differentially expressed genes. Optimization of the final signature included maximization of accuracy on the tissue of interest by selecting optimal thresholds of *P*-values and fold changes that were represented in a given fraction of the iterations. **b** Receiver-operating characteristic (ROC) curves for the prediction of training sets with the HER2 inhibitor Afatinib in breast cancer cell lines with inset accuracy and inset with area under the curve (AUC) values. A ROC AUC value of 1 represents perfect prediction and 0.5 represents random chance. **c** ROC curve for predicting HER2/Erbb2 patients based on the HER2i sensitivity score

As gene expression data has proven to have a robust capacity for predicting drug sensitivity,^5^ we developed a pipeline based on the stochastic sampling of gene expression data from publically available databases.^12^ The IRAPS (Fig. 1a) algorithm used gene expression data from 857 solid tumor derived cancer cell lines from the Cancer Cell Line Encyclopedia (CCLE),^12^ matched with drug sensitivity data from the Genomics of Drug Sensitivity in Cancer (GDSC).^20^ The reported sensitivity values were transformed into *z*-scores, defining “responders” defined as having *z*-scores of less than −1 and “non-responders” defined as having *z*-scores that were greater than 0. If multiple drugs were available for a given target, sensitivity values for both inhibitors were called and averaged after converting to *z*-scores. After 1000 iterations using the complete cell line set, the results were then transferred to a grid-search optimization algorithm for maximization of drug sensitivity prediction accuracy in the desired tumor type. This algorithm tested each threshold value for fold changes, *p*-values, and the fraction of times the gene was identified at each *p*-value/fold change threshold to determine which combination produced the optimal area under the curve (AUC) value for the receiver operator characteristic (ROC) curve, resulting in a final therapeutic target sensitivity.

For an initial training test of this pipeline, we sought to predict the sensitivity of breast cancer cells to dual HER2/EGFR inhibitors by using sensitivity data for neratinib and lapatinib. Optimization resulted in a 32 gene expression signature for HER2 inhibitor (HER2i, Table S1) sensitivity in breast cancer cell lines. The efficacy of this signature was confirmed in an independent testing set of cell lines treated with afatinib, yielding an AUC value of 0.96 with 94% accuracy (Fig. 1b). This signature identified ERBB3, which has been previously implicated in lapatanib response.^21^ To investigate if these cell line-derived signatures were relevant in patient cohorts, we applied our signature to TCGA breast cancer gene expression datasets^22^ to test the ability to identify patients within the HER2/ERBB2 breast cancer subtype and would likely be sensitive to HER2i. Though a population of the HER2/ERBB2 subtype may harbor innate resistance to HER2 inhibitors, or a subset of other subtypes may show response, the large enrichment within the HER2/ERBB2 subtype suggests the accurate prediction of responders to HER2 inhibition within breast cancer patients. While this signature did include ERBB2 and ERBB3, manually excluding these genes did not significantly alter prediction accuracy (Fig. S1). This analysis showed that our signature could identify the patients within the HER2/ERBB2 subtype with 90% accuracy (Fig. 1c), indicating our IRAPS approach could identify clinically relevant gene expression signatures from cell line drug sensitivity data.

### IRAPS accurately predicts patient survival following cisplatin treatment

**Fig. 2:**
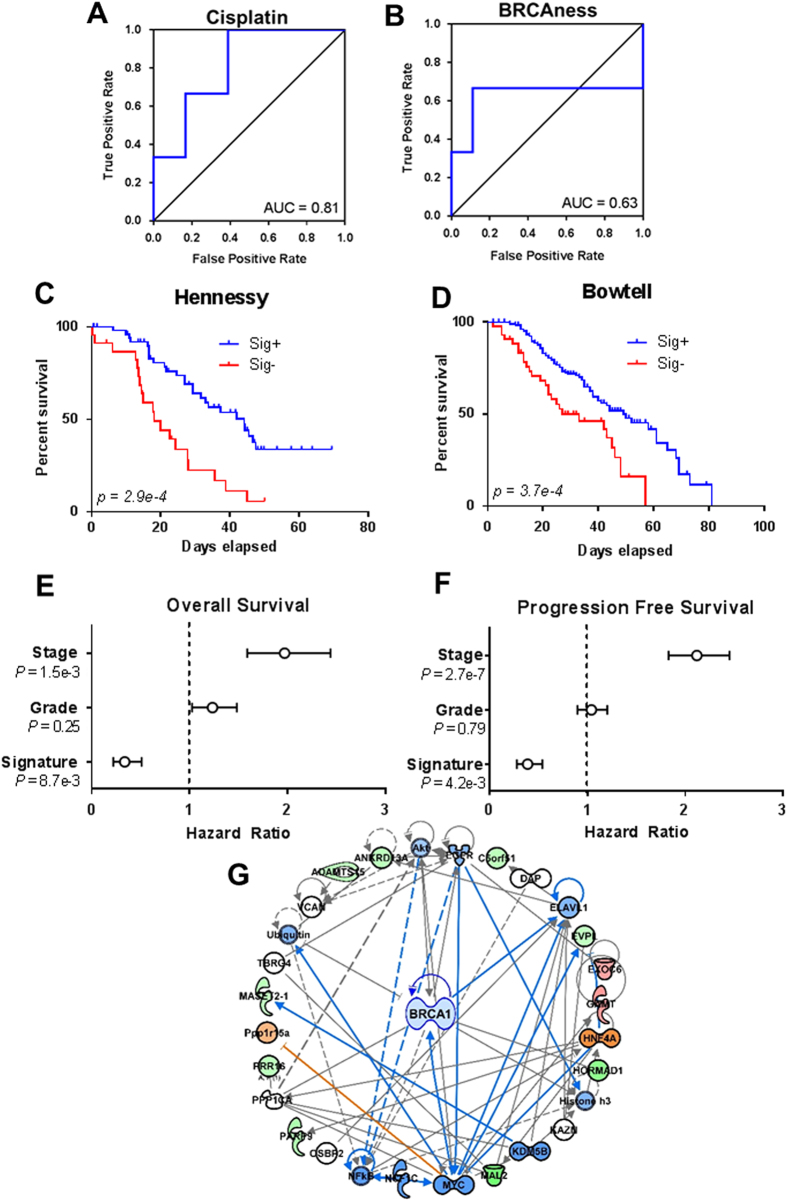
The cisplatin sensitivity signature that was generated from IRAPS predicts cisplatin-treated ovarian cancer patient survival independent of cancer stage and grade. **a** ROC curve based on the cisplatin sensitivity score using the cisplatin testing set that consisted of triple-negative breast cancer and ovarian cancer cell lines. **b** ROC curve for the same cells as in 2a, but based on the BRCAness sensitivity signature score. **c**, **d** Patient survival analyses in two independent cohorts of ovarian cancer patients that were treated with cisplatin were separated based on the cisplatin sensitivity score. Log-rank *p*-value is displayed that shows statistical significance. **e**, **f** High cisplatin sensitivity scores predict improved overall survival (**e**) and progression free survival (**f**) independent of stage and grade using a Cox proportional hazards model. **g** Ingenuity pathway analysis of the cisplatin sensitivity signature indicates a predicted down-regulation of *BRCA1*

To test the ability of IRAPS to predict sensitivity for a chemotherapeutic with a less-well defined profile, we generated a sensitivity signature for cisplatin. In order to have a sufficient sample size to allow for independent training and test groups, we optimized the signature for both serous ovarian cancer cell lines and triple-negative breast cancer cell lines. These cancers share many molecular commonalities, suggesting that there may be similar therapeutic vulnerabilities.^22^ The resulting 26 multigene expression signature (Table S2) was highly predictive of cisplatin sensitivity in the test data set, yielding an AUC of 0.81 (Fig. 2a).

Defects in the ability of cells to repair DNA double-stranded breaks contributes significantly to cisplatin sensitivity as a result of HR defects that are typically present in BRCA-mutant tumors or other phenotypically similar BRCA-like tumors.^23^ Thus, to evaluate the predictive ability of our IRAPS generated signature, we compared it to an established clinically-derived BRCAness signature.^24^ Our approach offered an improvement on BRCAness predictability, as evidenced by an increased ROC AUC (Fig. 2b). Next, to validate this signature in patient cohorts, we analyzed two independent ovarian cancer patient cohorts that were treated with cisplatin, with clinical-pathological characteristics of these cohorts are given in Table S3. We report that our cisplatin sensitivity signature predicted patient survival in both cohorts (Fig. 2c, d). Since both of these cohorts had gene expression profiled by microarray, we used TCGA samples profiled by both RNAseq and microarray to determine if the signature was sensitive to the technique used to profile samples. We found strong correlation between the score calculated through microarray and RNA sequencing suggesting the signature is rather insensitive to the profiling technique (Fig. S2). Moreover, the use of a Cox proportional hazards model suggested that this survival prediction was independent of both tumor stage and grade (Fig. 2e, f). These results compare favorably with previous signatures of platinum sensitivity derived from patients, with our cell line-derived signature yielding a multivariate hazard ratio of 0.33 (0.22–0.51) compared to hazard ratios of 0.30 (0.11–0.83) and 0.59 (0.34–1.01).^25^ This suggests that our signature derivation method can equal the accuracy of patient-derived signatures, enabling personalization of new chemotherapeutics in absence of large cohorts of patient data.

To understand what molecular changes the cisplatin sensitivity signature was detecting, we used Ingenuity Pathway Analysis and found that this signature predicted decrease in BRCA1 function, which may contribute to its predictive power (Fig. 2g). However, BRCA1 alone was not sufficient to predict responsive patients, as is evidenced by the low true positive rates in the BRCAness signature (Fig. 2b). Additional genes identified in our IRAPS signature, including the overexpression of DUSP6,^26^ and low level ERCC1 gene expression,^27^ have been previously identified as indicators of cisplatin sensitivity, and may therefore contribute to the accuracy of the signature.

### A PARP sensitivity signature improves identification of PARP-responsive tumors

**Fig. 3:**
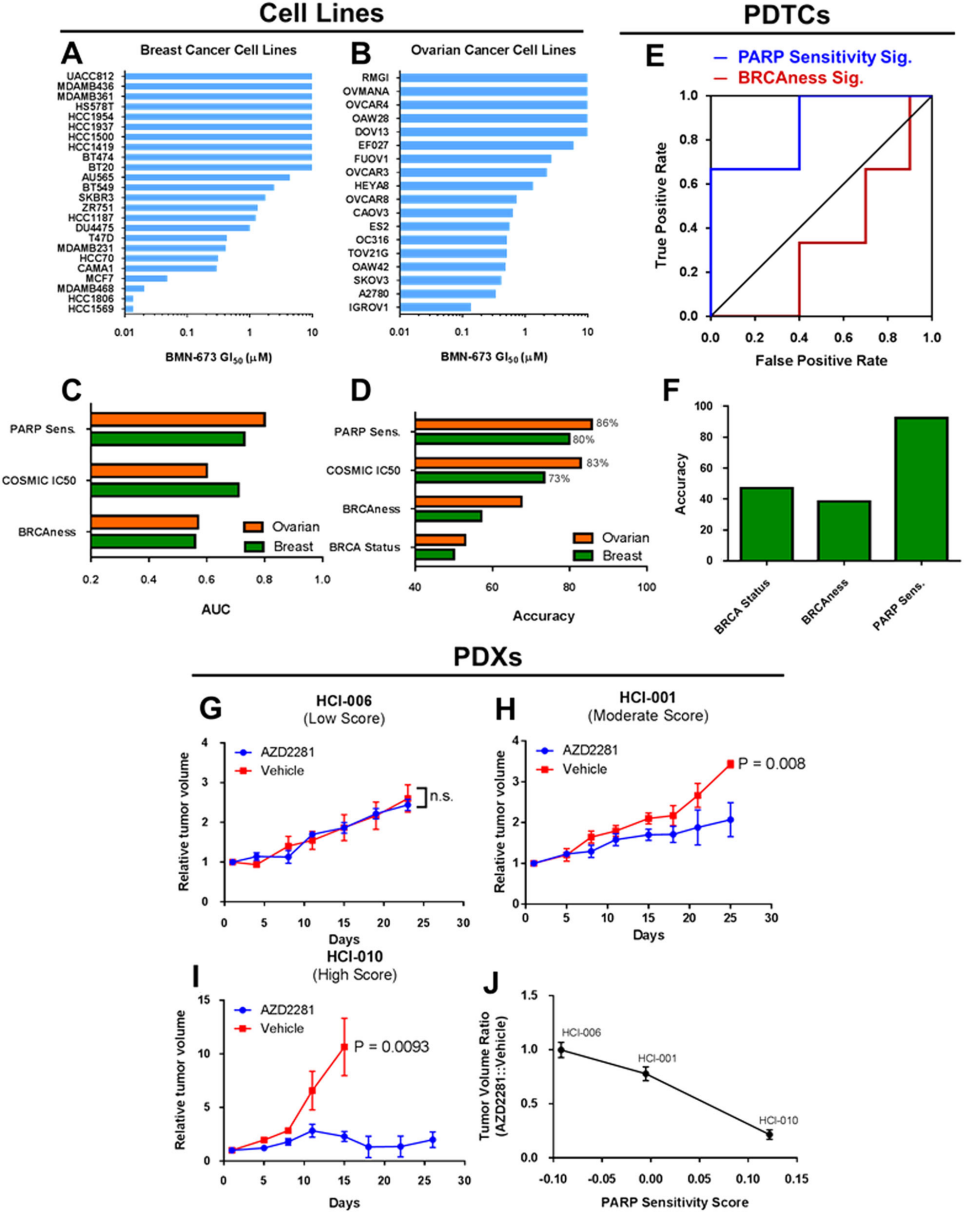
PARP sensitivity signature predicts response to PARP inhibitors in cell lines, patient-derived tumor cells (PDTCs), and patient-derived xenografts (PDXs). **a**, **b** Screening results for sensitivity to PARP inhibitor BMN-673 in breast (**a**) and ovarian (**b**) cancer cell lines. **c**, **d** AUC values from ROC curves (**c**) and overall accuracy (**d**) for prediction of sensitivity to the PARP inhibitor BMN-673 in ovarian and breast cancer cell lines determined based on BRCAness score and PARP sensitivity score, as well as by directly using the COSMIC IC_50_ values for AZD2281 (olaparib) and AG014699 (rucaparib) from which the PARP sensitivity signature was derived as an achievable upper bound. Overall accuracy was also analyzed based on *BRCA1/2* mutation status. **e** ROC curves for prediction of primary PDTCs^29^ response to BMN-673 shows PARPi sensitivity signature outperforms BRCAness signature. **f** Accuracy of predicting PARPi sensitivity for PTDCs based on PARPi sensitivity score, BRCAness score, and *BRCA1/2* mutation status. **g**–**i** Growth curves for breast cancer PDXs with low (**g**), moderate (**h**), and high (**i**) PARPi sensitivity scores following treatment with the PARP inhibitor AZD2281 QD at 50 mg/kg. **j** Ratio of tumor volumes in AZD2281-treated vs. vehicle controls. Ratios were calculated on day 15 and plotted as a function of the PARPi sensitivity score, demonstrating a strong negative correlation

PARP inhibitors have recently shown promise for the treatment of BRCA-mutant ovarian and breast cancers.^28^ However, it is unclear whether other patient populations may benefit from PARP inhibitors, as well as why the majority of BRCA-mutant patient tumors fail to respond to PARPi.^19^ We tested if a gene expression signature generated by IRAPS could improve patient selection for PARP inhibitors. For this analysis, we again optimized the algorithm for both ovarian and breast cancer cell lines by using sensitivity data for the PARP inhibitors AZD2281 (olaparib) and AG014699 (rucaparib) shown in Table S4. To generate an independent test set, we screened a panel of breast and ovarian cancer cell lines for sensitivity to the PARP inhibitor BMN-673 (Fig. 3a, b). Testing cell lines were excluded from the IRAPS pipeline. To determine an upper bound for signature accuracy, we used the PARPi sensitivity values from COSMIC to predict sensitivity in overlapping cell lines that were screened against BMN-673, and found that our gene signature performed equally well or better than this theoretical maximum attainable accuracy (Fig. 3c, d). To compare to more clinically relevant predictors, we compared our signature’s accuracy to either using the clinically-derived BRCAness gene signature^24^ as well as *BRCA1/2* mutation status and found our signature significantly outperformed both parameters (Fig. 3c, d). Of the evaluated breast cancer cell lines with mutations in *BRCA1* (MDA-MB-436 and HCC-1937) or *BRCA2* (HCC-1569, BT-20, BT-474, MDA-MB-361), only one (HCC-1569) was found to respond to BMN-673. Not only was this predicted by the signature, but also of the remaining five non-responding cell lines four were correctly identified as resistant, only misclassifying MDA-MB-436. Additional testing MDA-MB-436 response for olaparib (AZD2281) demonstrated sensitivity to this second agent, indicating this could be a drug-specific mechanism of resistance.

We next investigated the ability of the signature to predict response of a panel of primary PTDCs to BMN-673.^29^ The PARPi sensitivity signature again outperformed BRCAness signature as shown by the ROC curve (Fig. 3e), as well as accuracy relative to *BRCA1/2* mutation status (Fig. 3f). Notably, as observed with the breast cancer cell lines, the PARPi sensitivity signature accurately predicted the response of the two PARP-resistant *BRCA1/2* mutants that had gained additional mutations (STG316 with mutant *TP53BP1* ^30^ and VHI0179 with mutant *REV7*)^31^ reversing their HR defective phenotype. Finally, to test the accuracy of the signature to predict in vivo responses, we tested the response of three breast cancer PDX models with varying PARPi sensitivity scores (Fig. 3g–i). As expected, the low scoring PDX, HCI-006, did not respond to the PARP inhibitor AZD2281, whereas the moderate scoring PDX, HCI-001, gave a partial response, and the highest scoring PDX, HCI-010, having near complete tumor growth inhibition (Fig. 3i). Correlation of AZD2281 treated vs. control tumor volumes at day 15 displayed a strong negative correlation with the PARPi sensitivity score (Fig. 3j). This suggested that the PARPi sensitivity signature is capable of accurately predicting in vitro cell line responses and in vivo PDX responses to PARPi. Taken together, these results demonstrate that the PARPi sensitivity signature can accurately identify tumors responsive to PARPi both in *BRCA1/2*-mutant cohorts as well as broad panels of tumors.

### PARP sensitivity signature identifies PKCβ inhibitor enzastaurin as a novel PARPi synergizing agent

**Fig. 4:**
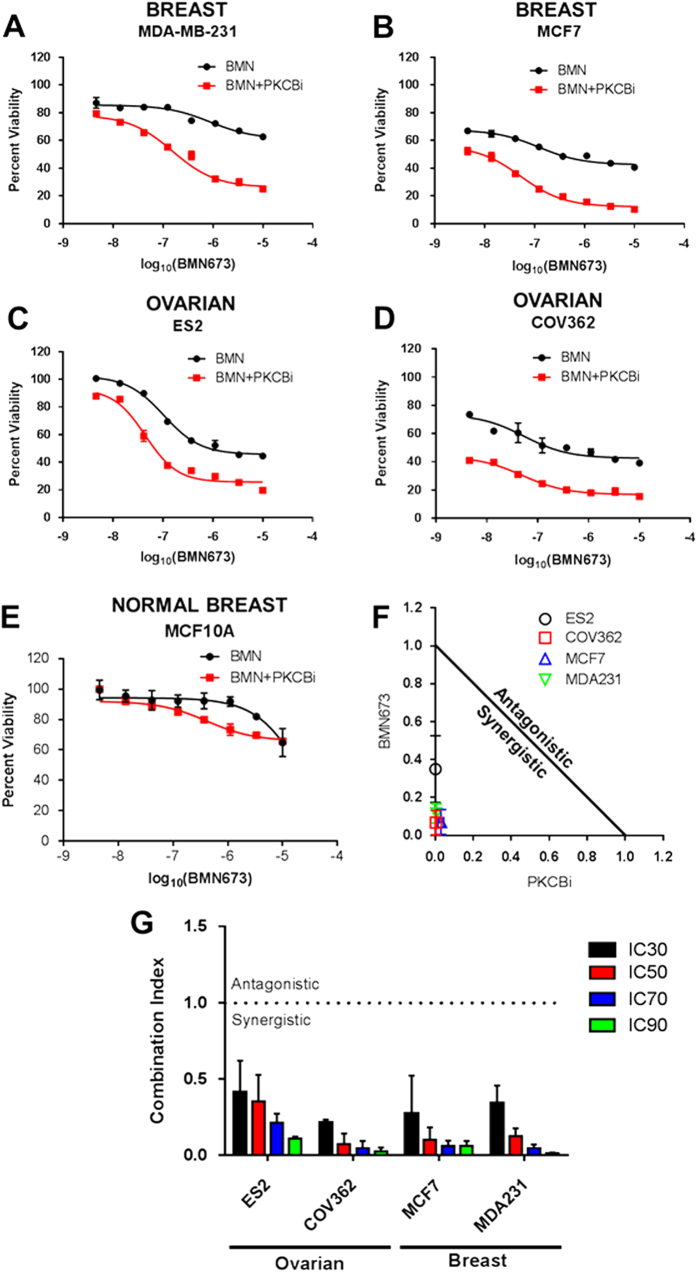
Identification of the PKCβ inhibitor Enzastaurin/LY317615 as a PARPi synergizing agent in breast and ovarian cancer cells. **a**–**e** Viability curves following BMN673 treatment with or without the PKCβ inhibitor LY317615 at a constant molar ratio of 1:3 BMN673:LY317615 for 5 days in the breast cancer cells **a** MDA-MB-231, and **b** MCF7, and in the ovarian cancer cell lines **c** ES2 and **d** BRCA-mutant COV362, as well as in the **e** non-transformed mammary epithelial cell line MCF10A. **f** Isobolograms calculated at IC_50_ values for cancer cell lines demonstrate synergism for all analyzed lines. **g** Combination indices demonstrate synergism between PARPi and PKCβi in a panel of ovarian and breast cancer cell lines across a range of inhibitor concentrations

Despite the promise of PARP inhibitor efficacy for the treatment of BRCA-mutated germline tumors, or in tumors that have a BRCAness phenotype, clinical trial objective response rates rarely exceed 50%.^28^ Thus, a clinical need remains for the improvement of methods to enhance PARP inhibitor efficacy. We hypothesized that if we were able to induce the PARPi sensitivity signature through pharmacological intervention, combinatorial therapy with PARP inhibitors may improve on PARPi efficacy. Therefore, we analyzed the PARPi signature using a pipeline developed by the Library of Integrated Network-based Cellular Signatures (LINCS) program termed “lincscloud”.^32^ This program collects gene expression data following numerous chemical and genetic perturbations, allowing for the identification of possible inducers of the PARPi sensitivity signature. A top hit amongst molecules that were involved in clinical testing was an inhibitor of PKCβ. Initial testing of PKCβ inhibition in combination with the PARP inhibitor BMN673, revealed an enhanced PARP inhibitor response for both the triple negative breast cancer cell line MDA-MB-231 (Fig. 4a), and the luminal breast cancer cell line MCF7 (Fig. 4b). Not only did PKCβ inhibition synergize with PARPi in breast cancer cell lines, the combinatorial efficacy was also evidenced for the BRCA wildtype ES2 (Fig. 4c) and BRCA-mutant COV362 ovarian cancer cell lines (Fig. 4d). This sensitization was greatly diminished in the non-tumorigenic MCF10A human mammary epithelial cell line (Fig. 4e). Isobolograms determined from IC_50_ values indicate that PARP and PKCβ inhibition synergized in all tumorigenic cell lines (Fig. 4f). Moreover, quantification of synergism using the Chou-Talalay combination index method, where values that were less than 1 represented synergistic combinatorial therapies (see ref.^51^), demonstrated that this combination was synergistic in all evaluated cell lines across a wide range of concentrations (Fig. 4g). As this combination induced synergy in both BRCA-mutant and wild type cell lines with no dependency on basal sensitivity, this combination may be a viable treatment option to enhance efficacy in BRCA-mutant patients or cohorts predicted by the PARPi sensitivity signature. Conversely, we found that Cucurbitacin I was predicted to reverse the PARPi sensitivity signature and thus tested to see if it would be antagonistic with PARP inhibitors (Fig. S3). As expected, this combination was found to be antagonistic in ES2 cells with less significant interaction between the drugs in COV362 cells, possibly because their BRCA mutation is too strongly sensitizing to PARP inhibitors to be reversed by chemical modulation.

**Fig. 5:**
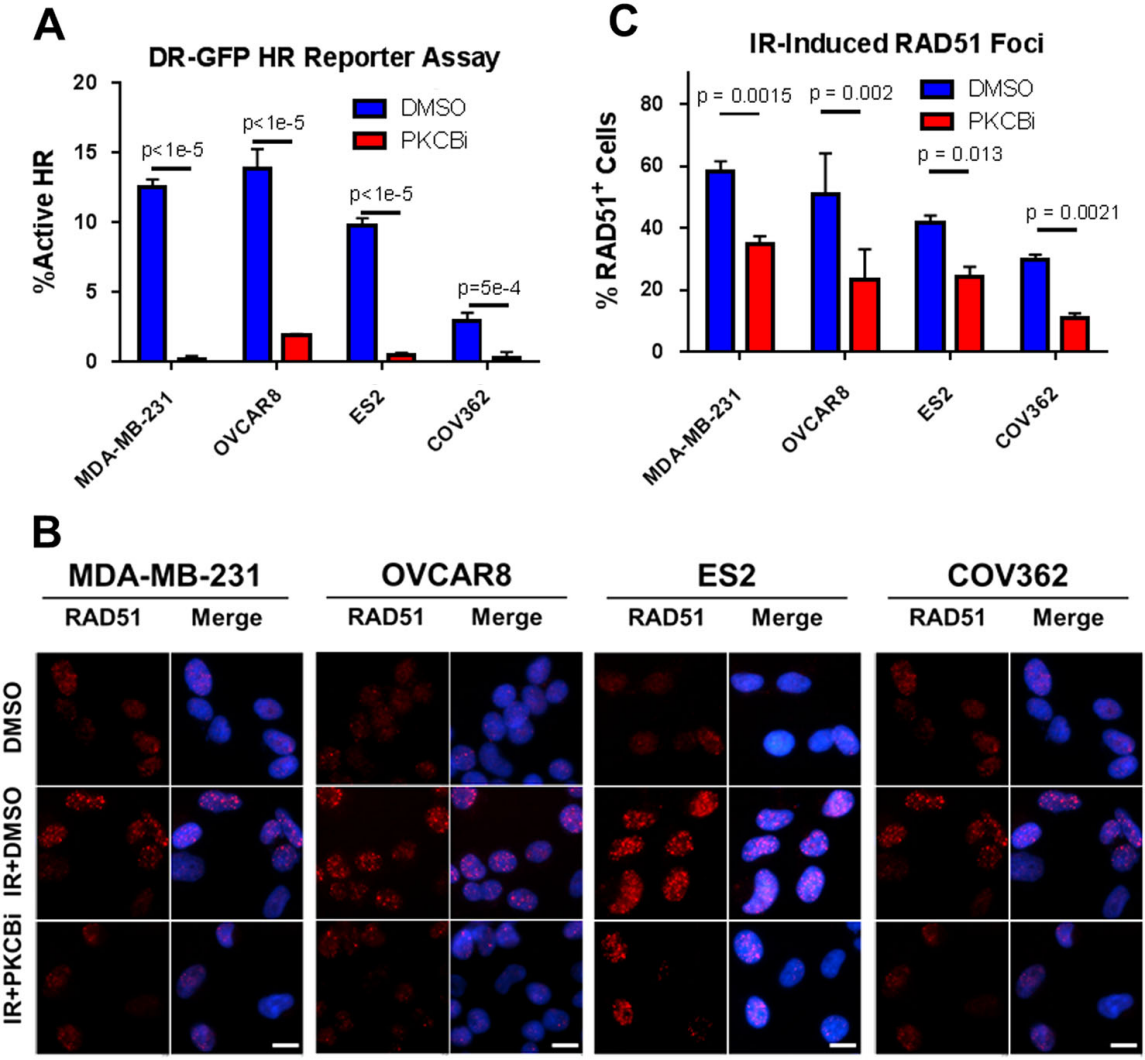
PKCβ inhibition induces deficiencies in homologous recombination. **a** DR-GFP homologous recombination (HR) reporter assay, where GFP induction is induced in cells with active HR following dual transfection with the DR-GFP and the pCBA-SceI. The day after transfection, cells were treated with either 5 μM LY317615 or DMSO vehicle control for 48 h. The percentage of cells with active HR was calculated as the percentage of DR-GFP positive cells normalized to percentage GFP positive cells transfected with an equimolar amount of pEGFP-C1. **b** Reduction in radiation-induced Rad51 foci formation shows inhibited HR. Cells were pre-treated with 5 μM LY317615 for 4 h, irradiated at 5 Gy, and then allowed to recover for 4 h before immunostaining for Rad51 (*red*) and nuclei (*blue*). Scale bar = 10 μm. **c** Quantification of images in 5b, when considering that cells with more than 10 Rad51 foci are deemed as being positive

Since PARP inhibitors should preferentially target cells with defective HR, we hypothesized that PKCβ blockade was impairing HR. To test this, we used the direct repeat GFP (DR-GFP) reporter assay in which cells with a functional HR machinery will recombine the plasmid and express the GFP protein that is detectable by flow cytometry.^33^ As expected, the BRCA-mutant COV362 cell line had the lowest latent levels of HR. However regardless of the cell lines tested, HR activity was nearly completely abrogated with PKCβ inhibition (Fig. 5a). We verified this result by testing the ability of these cells to form RAD51 foci following irradiation, and found that RAD51 foci induction was reduced by over 50% (Fig. 5b, c). Comparing effects on the DR-GFP reporter assay relative to RAD51 foci formation as an indication of the extent of HR blockade suggested that PKCβ plays a role in the formation of RAD51 foci and in the later stages of HR.^34^ Moreover, this inhibition of HR was markedly reduced in non-tumorigenic MCF10A (Fig. S4). Taken together, not only was the IRAPS pipeline capable of generating a robust signature that accurately predicted PARPi sensitivity, but this resulting signature was also capable of predicting clinically relevant molecules that block HR and synergize with PARP inhibitors.

### Prediction of BRCAness-targeting drugs

**Fig. 6:**
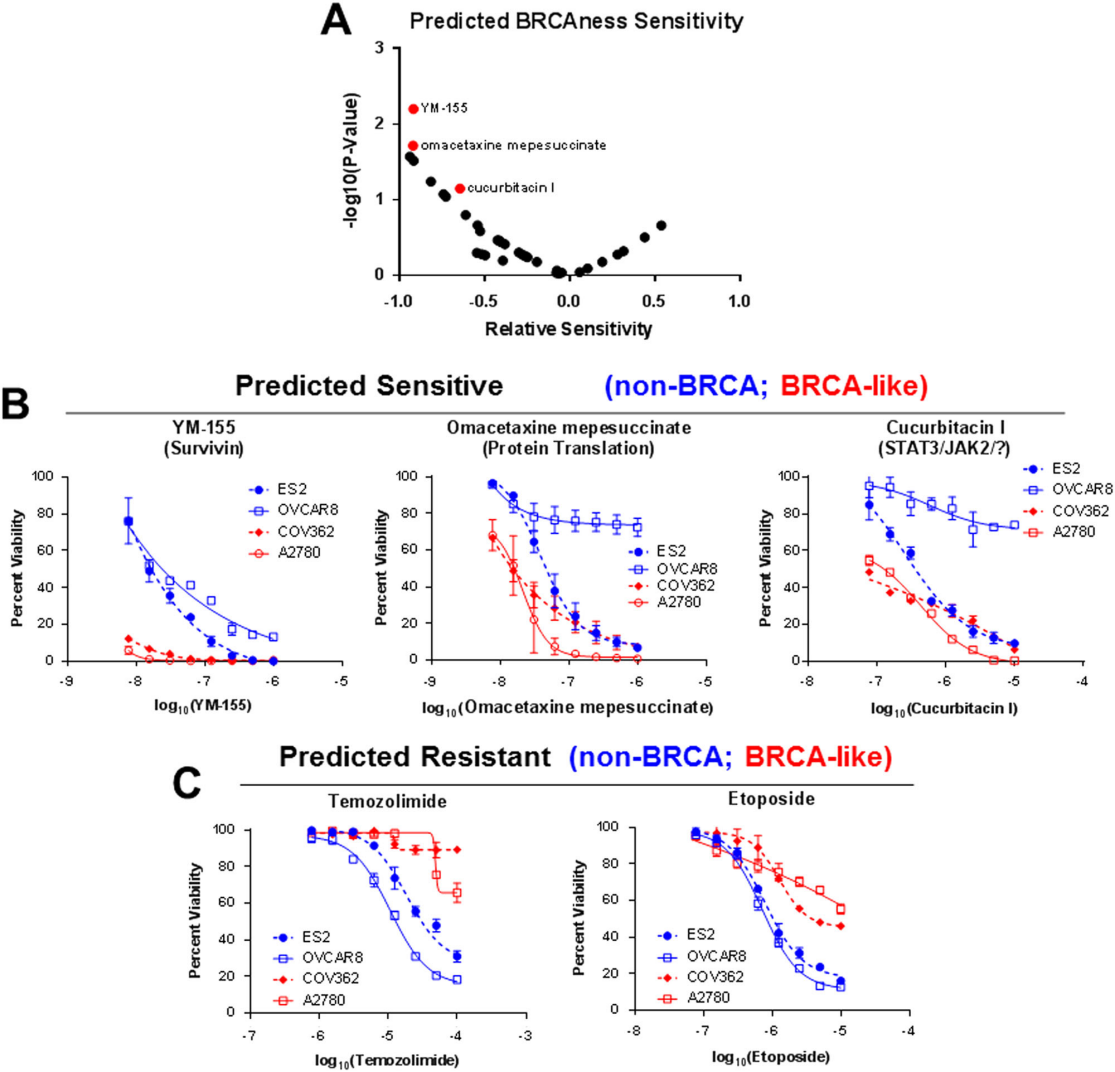
Use of the BRCAness signature to predict for targeted therapies reveals that the survivin inhibitor YM-155 specifically targets BRCA-like cells. **a** Volcano plot of compounds predicted to target BRCA-like cells based on the BRCAness signature score. **b** Viability curves following 72 h of treatment with identified BRCAness-targeting molecules for *BRCA1* mutant COV362, BRCA-like A2780, and non-BRCA-like ES2 and OVCAR8. **c** Viability curves following 72 h of treatment with drugs that BRCA-like cells are predicted to be resistant to

Since the BRCAness signature was not predictive for either cisplatin or PARP inhibitors, we questioned what drugs may potentially target BRCA-like cell lines. To investigate this, instead of classification based on drug sensitivity to ascertain differentially regulated genes, we applied the BRCAness signature to assign a BRCAness phenotype to cell lines, and then used the CTRPv2 database to predict differential drug sensitivity. After excluding drugs with area under the response curve values of larger than 10, which is indicative of a general lack of cytotoxicity, we selected three compounds for further testing (Fig. 6a). These compounds were screened in four cell lines that were excluded from the initial analysis: BRCA mutant COV362 cells, BRCA-like CAOV4 cells, non-BRCA-like ES2 cells, and non-BRCA-like OVCAR8 cells. All three of the predicted BRCAness targeting drugs showed preferential toxicity in the BRCA-like cells (Fig. 6b). As expected, drugs that BRCA-like cells were predicted to be resistant to displayed diminished efficacies (Fig. 6c). While all predicted drugs showed some preferential inhibition of cells with a high BRCAness score, the survivin inhibitor YM-155 exquisitely targeted BRCA-like cells at nanomolar IC_50_ amounts. Combination of YM-155 with the standard of care chemotherapeutics, cisplatin and paclitaxel, in BRCA-like ovarian cancer may be a promising treatment approach since early clinical trials in advanced non-small cell lung cancer have shown a favorable safety profile for YM-155 when combined with cisplatin and paclitaxel.^35^

## Discussion

Identification of predictive and prognostic biomarkers has had a profound effect on the treatment and prognoses of various cancers and of their sub-types. For example, estrogen receptor and HER2 immunoreactivity of breast cancers are used to guide their treatment, and ultimately improve on their outcomes. However, response to many therapeutics with more complex mechanisms of action cannot be predicted from a single molecular marker and thus require development of systems-level signatures for optimal prediction of patient response. The capacity for improving cancer management through the use of drug sensitivity gene expression profiles, beyond the standard clinicopathologic variables, is necessary to advance the era of individualized cancer care. The rapidly decreasing price of gene expression microarrays and RNAseq makes it plausible to integrate this technology as a companion diagnostic into clinical trials for the eventual individualization of patient care. While results from gene expression profiling studies are changing how patients are being managed in the clinic,^8–10^ and guiding which therapies individuals should receive,^36–38^ optimal approaches to generate relevant signatures are unknown.

Here, we demonstrate the utility of our novel IRAPS algorithm for the generation of tissue-specific drug sensitivity gene expression signatures from cancer cell line gene expression and drug screening data. After demonstrating the efficacy of this algorithm on HER2i, we verified its potential with two therapeutics: cisplatin and PARP inhibitors. Both of these therapeutics are thought to best target tumor cells with deficiencies in HR DNA repair, such as those with *BRCA1/2* mutations. There has been particular excitement with the development of PARP inhibitors for treatment of BRCA-mutant patients, as the specific targeting of HR-defective cancer cells not only offers a powerful treatment option, but also mitigates side-effects by primarily targeting the cancerous cells with HR deficiencies as opposed to normal cells.^39^ Initial trials in treating BRCA-mutant patients were so successful that two PARP inhibitors were given breakthrough therapy designation by the FDA for accelerated approval.

Despite this promise, analysis of recent clinical trials demonstrates a clear need for improved identification of patient populations to treat with PARP inhibitors. Across five independent clinical trials, the majority of BRCA-mutant patients still fail to respond to PARP inhibitor treatment, with an average objective response rate of 47%, compared to 20% in wild-type BRCA patients.^19^ The non-responding BRCA-mutant patients may be explained by phenomena including concurrent mutations such as loss of either *TP53BP1* ^30^ or *PTEN* ^40^ that can lead to restoration of HR-mediated DNA repair. The PARPi sensitivity signature correctly identified the only *BRCA1/2* mutant cell line found to respond to BMN-673, as well as 4/5 BMN-673 resistant cell lines with *BRCA1/2* mutations. Similarly, both *BRCA1/2* mutant PDTCs were correctly predicted by the signature to not respond to BMN-673. This ability to accurately predict response within *BRCA1/2* mutant tumor cells indicates future that trials implementing the signature could be performed on *BRCA1/2* cohorts already being treated with PARP inhibitors before expanding to larger cohorts.

Conversely, it is also known that a wide variety of mutations outside of *BRCA1/2* can lead to HR defects, such as *RAD51* or *BLM*, which may partially explain the 20% of responders without mutations in *BRCA1/2*.^41^ Tumors with HR deficiencies arising from sporadic mutations or silencing of genes that are essential for HR have been termed BRCA-like.^42^ Not only are BRCA-like tumors associated with PARP inhibitor sensitivity, but they have also been shown to have better response to platinum therapeutics such as cisplatin.^24^ Importantly, with nearly a quarter of a million new cases of breast cancer in the United States annually,^43^ this 20% of responding patients with wild-type *BRCA1/2* represents nearly 50,000 patients, four-fold more than the sum of all *BRCA1/2*-mutation carriers. The PARPi sensitivity signature could serve as an important tool to identify these patients. Within the breast cancer cell lines we screened lacking *BRCA1/2* mutations, the PARPi sensitivity signature predicted to five to be sensitive (HCC-1806, CAMA1, MCF7, MDA-MB-231, and BT-549). Of these five predicted responders, four were found to be sensitive to BMN-673, with the 5th (BT-549) showing a moderate response. Thus, this PARPi sensitivity signature fulfills an urgent clinical need for improved prediction of PARP inhibitor sensitivity to not only prevent treatment of *BRCA1/2*-mutant non-responding patients, but also to identify the substantial number of patients without BRCA mutations who could potentially benefit from treatment.

To further illustrate the power of these predictive signatures, we demonstrate their utility for prediction of potential synergizing agents using the lincscloud database. By comparing our signature with the gene expression changes induced by thousands of chemical perturbations, we were able to identify potential synergistic agents that could induce our PARPi sensitivity signature. Several of these molecules blocked PKCβ, and ensuing experiments demonstrated that PKCβ inhibition with Enzastaurin/LY317615 strongly synergizes with PARP inhibitors (Fig. 4). Follow-up studies demonstrated that treatment with Enzastaurin/LY317615 was sufficient to block HR in a wide variety of cancer cell lines, but had minimal effect on non-tumorigenic MCF10A cells (Fig. 5, Fig. S4), suggesting that elevated PKCβ activity may be required for the maintenance of HR activity in cancer but not normal cells. To our knowledge, this is the first reported evidence of PKCβ being associated with HR in human cell lines.

Since sensitivity to both PARP inhibitors and cisplatin are thought to correlate with the BRCAness phenotype, we additionally compared our IRAPS-derived signature to a clinically-derived signature of BRCAness.^24^ Notably, both our signatures outperformed this clinical BRCAness signature, which led us to question what molecules may best target the BRCAness tumor phenotype. The most effective of these was the survivin inhibitor YM-155 (Fig. 6). This molecule offers an attractive therapeutic option in BRCA-like ovarian cancer as combination of YM-155 with the standard of care chemotherapeutics, cisplatin and paclitaxel, has shown a favorable efficacy and safety profile in early advanced non-small cell lung cancer clinical trials.^35^ Previous studies have demonstrated not only that loss of *BRCA1* can lead to an up-regulation of survivin,^44^ but also that survivin is a key activator of the HR-mediated DNA repair pathway.^45^ Taken together, these findings could suggest that survivin up-regulation is compensatory mechanism following loss of BRCA function, resulting in the observed high sensitivity of BRCA-mutant and BRCA-like cell lines.

Collectively, by summarizing large-scale genomic data, this work has allowed for complex datasets to be placed in a biological context that may be used to provide molecularly tailored treatment recommendations, and also provide a foundation for the discovery of synergistic therapies that could be further tested or used for individualized care. The predictive algorithm that we have generated, may not only prove useful in the clinical setting for guiding treatment decisions, but may also be generalized to answer additional questions from other human diseases that require similar interrogation.

## Materials and methods

### Iterative resampling analysis to predict sensitivity

Gene expression data for 857 solid-tumor derived cancer cell lines was downloaded from the CCLE database.^12^ Data were log_2_ transformed, quantile-normalized, and median polished on a per-tissue type basis. This data was then combined with drug sensitivity data from either the GDSC dataset (PARP inhibitors, cisplatin),^20^ or from the Cancer Therapeutic Response Portal v2 (HER2 inhibitors).^46^ To determine a list of differentially expressed genes, a random selection of 50% of available cell lines were sampled and sensitivity values (the half maximal inhibitor concentration (IC_50_) for GDSC and AUC for the drug dose response curve for CTRPv2) converted into z-scores, which were used to define a class of “responders” (*z*-score < −1) and “non-responders” (*z*-score > 0). To improve predicative capacity, the average *z*-score of two inhibitors was used to define sensitivity for PARP (rucaparib and olaparib) and HER2 inhibitors (lapatanib and neratanib) enforcing that responders both had a minimal *z*-score of −0.5 and both non-responders had *z*-scores greater than zero. This resulted in approximately 20% of cells being classified as responders, 50% as non-responders, and 30% falling into neither category. Specifically, approximately 30 responders/110 non-responders were identified for HER2 inhibitors per iteration, 25 responders/70 non-responders for cisplatin, and 15 responders/50 non-responders PARP inhibitors. For each panel a sub set of samples was excluded for testing the signatures: for testing the HER2 signature two samples were excluded for HER2 inhibitors plus 16 additional that were tested for afatinib but not lapatanib and neratanib, for testing the cisplatin signature half of basal breast cancer cell lines were excluded, and for testing the PARP inhibitor signature all cell lines screened against BMN-673 were excluded from training. This process was repeated 1000 times by selecting a different random population each time to generate 1000 lists of differentially expressed genes. To refine these gene expression datasets that were generated across all solid tumors, we used a grid-search optimization algorithm to maximize the accuracy of prediction in the desired tumor types across all combinations of gene fold changes, *p*-values, and conservation across the multiple iterations—that is the frequency a gene was found to be altered at a given *p*-value and fold change threshold. At each threshold, the average gene fold change for each gene meeting the specified criteria was used as the gene weight. For example, for *p* = 1e − 4, fold change = 1.25, and conservation value of 0.8 the signature would consist of all genes that had *p*-values < 1e − 4 and fold changes >1.25 in at least 800/1000 iterations, with weights consisting of the average fold changes across all runs for these genes. Signature scores were determined by calculating the correlation coefficient between gene weights within the signature and gene expression levels for that gene within a given sample.^47^ For the HER2 inhibitor signature this was breast cancer, for cisplatin a combination of endometrial and basal breast cancer was used to preserve the ovarian cancer lines for testing, and for the PARP inhibitors ovarian and breast cancers were used. The derived signatures were tested against afatanib-treated breast cancer for the HER2 signature, ovarian cancer and the remaining half of basal breast cancer cell lines for cisplatin, and BMN-673-treated breast and ovarian cancer cell lines for the PARPi signature. All sensitivity signatures are included in Tables S1, S2, and S4.

### Patient survival analysis

The cisplatin sensitivity signature was used to predict patient prognosis for ovarian cancer patients that were treated with cisplatin in two independent cohorts, Hennessy^40^ and Bowtell.^48^ Signature scores were calculated following quantile normalization. To generate Kaplan–Meier curves, patients were divided based on the maximization of statistical difference in signature scores between the two groups. To account for stage and grade as covariates, a Cox proportional hazards model was used.

### Cell line screening of BMN-673 sensitivity

Drugs were three-fold serial diluted for seven dilutions in DMSO at 1000× concentration stocks. Aliquots of the diluted stocks were stored in deep-well “master plates” in −20 °C. Cancer cell lines involved in this assay were verified by short tandem repeat analysis (CCSG Characterized Cell Line Core in MD Anderson Cancer Center). Cell lines were maintained in their optimal growth medium (with 5% FBS) and seeded in 96-well plates at 2500 cells/100 μL/well for 24-h incubation prior to be changed into the medium containing 2% FBS for overnight incubation. Serial diluted drug stocks were added to each well to make 1/1000 final concentration for additional 7-day incubation. DMSO at 0.1% without any drugs was used as controls. Triplicates were performed for testing each concentration. Cell viability was determined by using the Cell Titer Blue Cell Viability Assay (5 μL of the reagent/well) and measured at 530Ex/604Em. Cell viability was defined by GI_50_ concentration, defined as the concentration required to slow cell growth by 50%, calculated according to a cell viability curve.

### Patient-derived tumor cells

The sensitivity of PTDCs to BMN-673, along with relevant gene expression and mutation data, was acquired from the work of Bruna and colleagues.^29^ Data for the PDX models HCI-001, HCI-006, and HCI-010 were excluded from analysis as they were used for in vivo testing. For models that had multiple replicates, the earliest passage explant matched with the nearest passage of gene expression data was analyzed.

### Patient-derived xenografts

Patient-derived breast cancer xenografts (HCI-001, HCI-006, and HCI-010) with matching RNAseq data (GSE32532) were acquired from the University of Utah Patient-Derived Xenograft Repository and implanted as described into athymic nu/nu mice.^49^ After reaching a volume of 100 mm^3^ treatment was initiated via intraperitoneal injection with 50 mg/kg AZD2281 or with 40% PEG400 as the vehicle control. Treatments were given QD for 28 consecutive days as previously described.^50^ The study made use of ten mice per treatment arm, and tumor volumes were tracked with calipers in order to calculate tumor volumes as (length × width2)/2. End-point mice were sacrificed by carbon dioxide inhalation. The treatment of animals were done in accordance with Institutional Animal Care and Use Committee protocols at MD Anderson Cancer Center. Tumor volumes were reported as their mean ± SEM.

### PARP inhibitor synergizing agents

In order to predict drugs that may sensitize cells using our PARP sensitivity signature, we used the lincscloud (http://www.lincscloud.org) database that compares our signature with thousands of chemical perturbations. We obtained a ranked list of candidate molecules, and then verified the top hits in breast cancer and ovarian cell lines that were available to us. Based on this analysis, we identified PKCβ inhibition as a top hit, and tested the PKCβ Enzastaurin (LY317615) in combination with the PARP inhibitor BMN-673 using a molar ratio of 3:1, respectively. Cells were treated for 5 days with either LY317615, BMN-673, or in combination before we analyzed cell viability with PrestoBlue (Invitrogen) per the manufacturer’s instructions. Each independent replicate was performed with two technical replicates. Combination indices were calculated using CompuSyn software,^51^ where values under one represented synergism and values over one represented antagonism.

### HR assays

HR activity was assessed by two separate approaches. First, we utilized the DR-GFP reporter assay as previously described.^33, 40^ Briefly, cells were transfected with equimolar amounts of either a combination of DR-GFP and pCBASceI (gifts from Dr. Maria Jasin; Memorial Sloan-Kettering Cancer Center, New York, NY) using Lipofectamine 3000 (Invitrogen), which induces green fluorescence in cells with an active HR machinery, or by using a GFP-expressing plasmid (pEGFP-C1) as a transfection efficiency control. The day following transfection, cells were treated with 5 μM LY317615 and then harvested 48 h later for flow cytometry analysis. GFP positive cells were gated based on SSC-FITC scatter plots. Percentages of cells with active HR were calculated as 100× (% DR-GFP^+^ cells)/(% pEGFP^+^ cells), with at least 50,000 cells having been analyzed per condition. We additionally analyzed the ability of cells to form RAD51 foci following gamma irradiation. Cells were pre-treated with 5 μM LY317615 for 4 h before treatment with 5 Gy radiation. After recovering for 4 h, cells were stained for RAD51 foci as previously described^52^ with an anti-RAD51 antibody (sc8349, Santa Cruz Biotechnology). Cells were imaged and analyzed by fluorescence microscopy (Eclipse TE2000E, Nikon) and automatically quantified in a custom-written MATLAB algorithm (MathWorks). Cells with more than ten foci were counted as positive, with a minimum of 50 cells scored across three independent experiments for at least 150 cells in total per condition.

### Prediction of drugs targeting the BRCAness signature

Prediction of BRCAness-targeting drugs was carried out using drug sensitivity data from the CTRPv2 database.^46^ For this analysis, expression data of ovarian cancer cell lines (excluding those that were used for testing) was extracted and quantile normalized before calculating a BRCAness score based on a previously established BRCAness signature.^24^ Cells with BRCAness scores that were greater than 1 standard deviation above the average were considered positive, and cells with a below average score were considered negative. The drug sensitivity data for these cell lines were extracted and used to identify drugs that specifically targeted cells with high levels of BRCAness. Drugs were selected that were both selective and had high toxicities in the BRCA-like group, which were defined as area under response curve values of 9 or less, and tested in COV362 (*BRCA1* mutant), A2780 (BRCA-like), ES2 (non-BRCA-like), and OVCAR8 (non-BRCA-like) cells. Additionally, molecules that BRCA-like cells were predicted to be resistant to were also tested. For these studies, cells were plated at 5000 cells/well in a 96-well plate and treated with serial dilutions of specified drugs for 72 h before viability quantification with PrestoBlue.

### Statistical analysis

Unless otherwise noted, statistical significance was determined by either using a student *t*-test or a two-way ANOVA with post-hoc analysis from triplicate independent experiments. All data are reported as the mean ± standard error of the mean (SEM).

## Electronic supplementary material

Supplementary Information

Supplementary Table1

Supplementary Table2

Supplementary Table3

Supplementary Table4
