# Supplementary material for: Improved prediction of PARP inhibitor response and identification of synergizing agents through use of a novel gene expression signature generation algorithm
Source: NPJ Syst Biol Appl. 2017 Mar 6;3:8. doi: 10.1038/s41540-017-0011-6 (PMC5445594; doi:10.1038/s41540-017-0011-6)
Supplement: Supplementary file 1 — Supplementary Information [file 41540_2017_11_MOESM1_ESM.pdf]

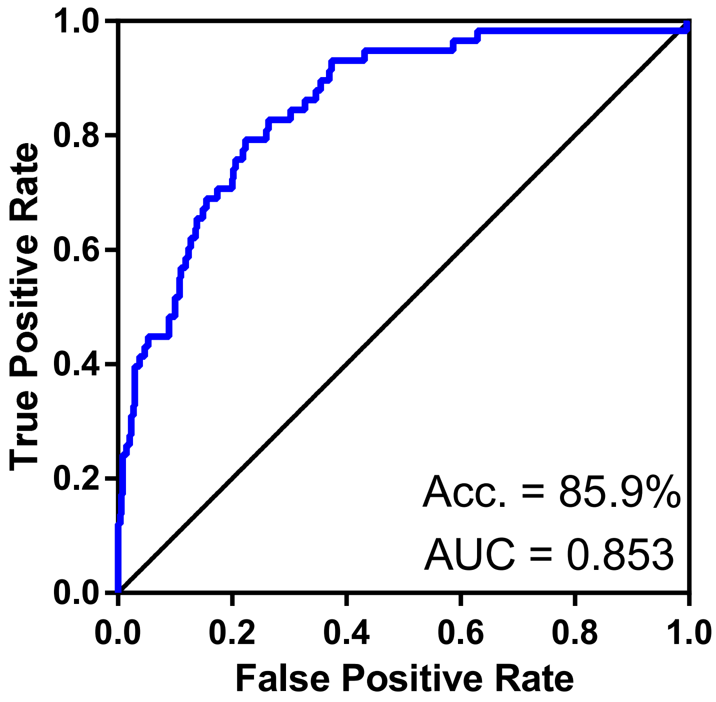

Figure S1. The HER2 inhibitor signature predicts independent of ERBB2 and ERBB3 expression.

After excluding ERBB2 and ERBB3 from the HER2 inhibitor sensitivity gene expression, results were re-calculated and showed minimum deviation from the full signature indicating these two genes are not largely responsible for the accuracy of the signature.

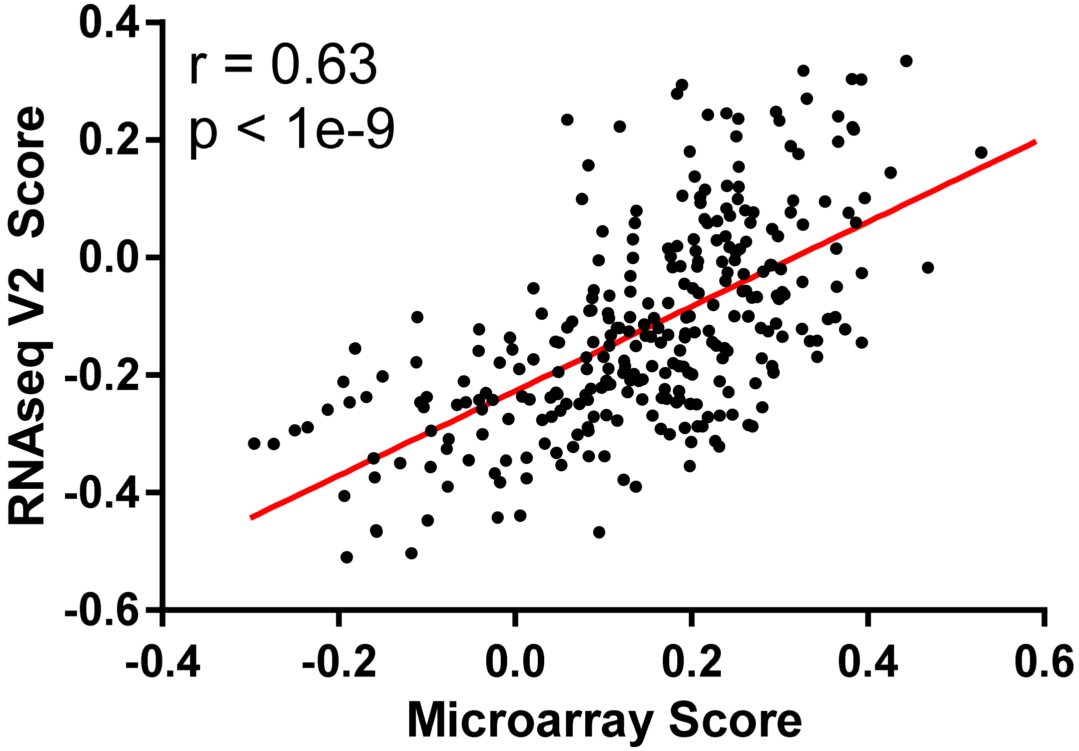

Figure S2. Gene expression signature score is not dependent on profiling method.

Cisplatin sensitivity scores were calculated for ovarian cancer patients from the TCGA that were transcriptionally profiled both by RNAseq V2 (Illumina HiSeq) and microarray (Affymetrix U133A). Both scores show strong correlation, indicating the approach is largely independent of profiling approach.

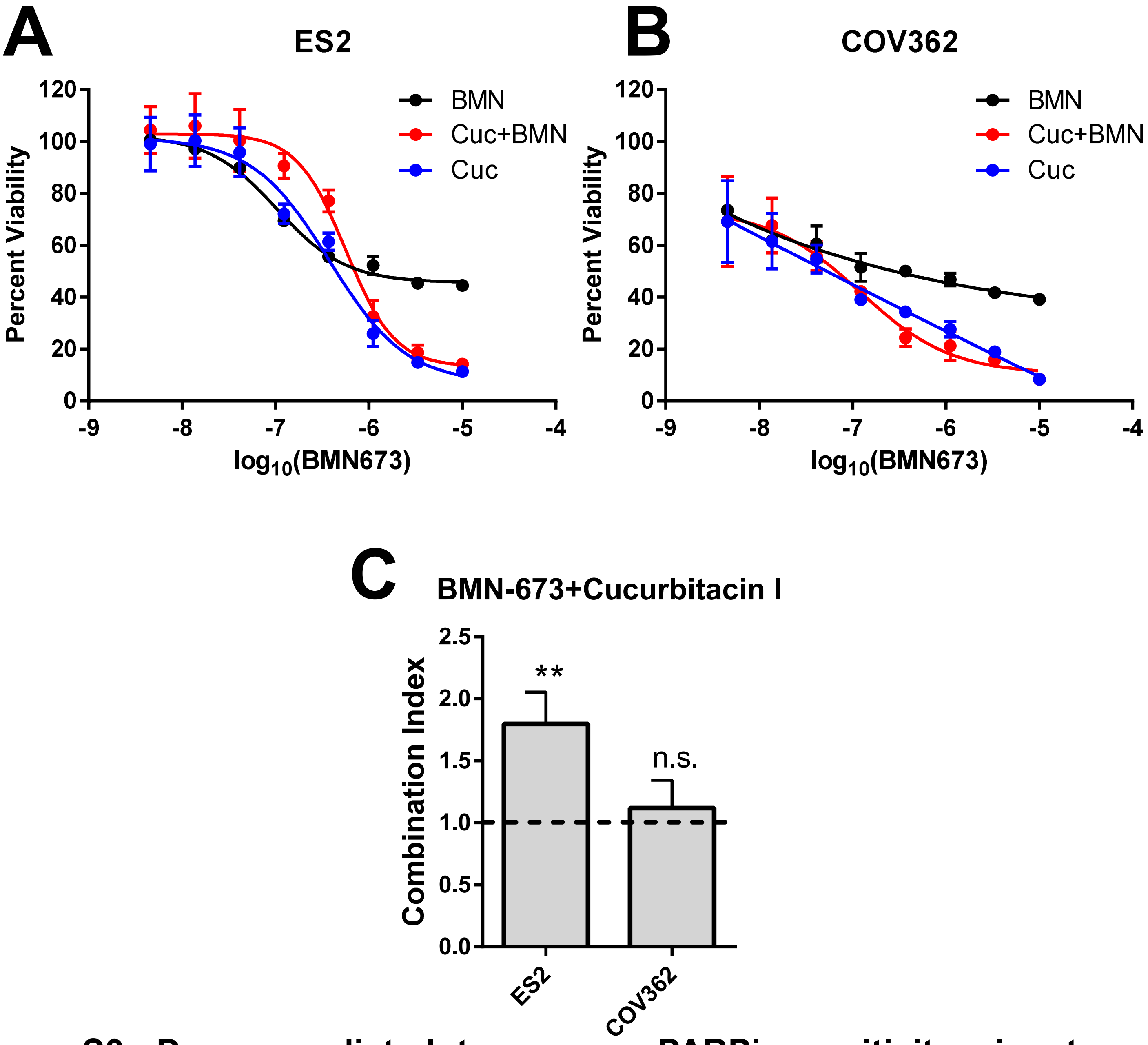

Figure S3. Drugs predicted to reverse PARPi sensitivity signature are antagonistic.

A Dose-response curves for cells treated with BMN-673 in combination with Cucurbitacin I, which was predicted reverse the PARPi sensitivity signature.

B Quantification of combination index from IC_{50} values shows significant antagonism (combination index > 1) in ES2 ovarian cancer cells and no interaction in COV362. * indicates significantly antagonistic, $p<0.01$.

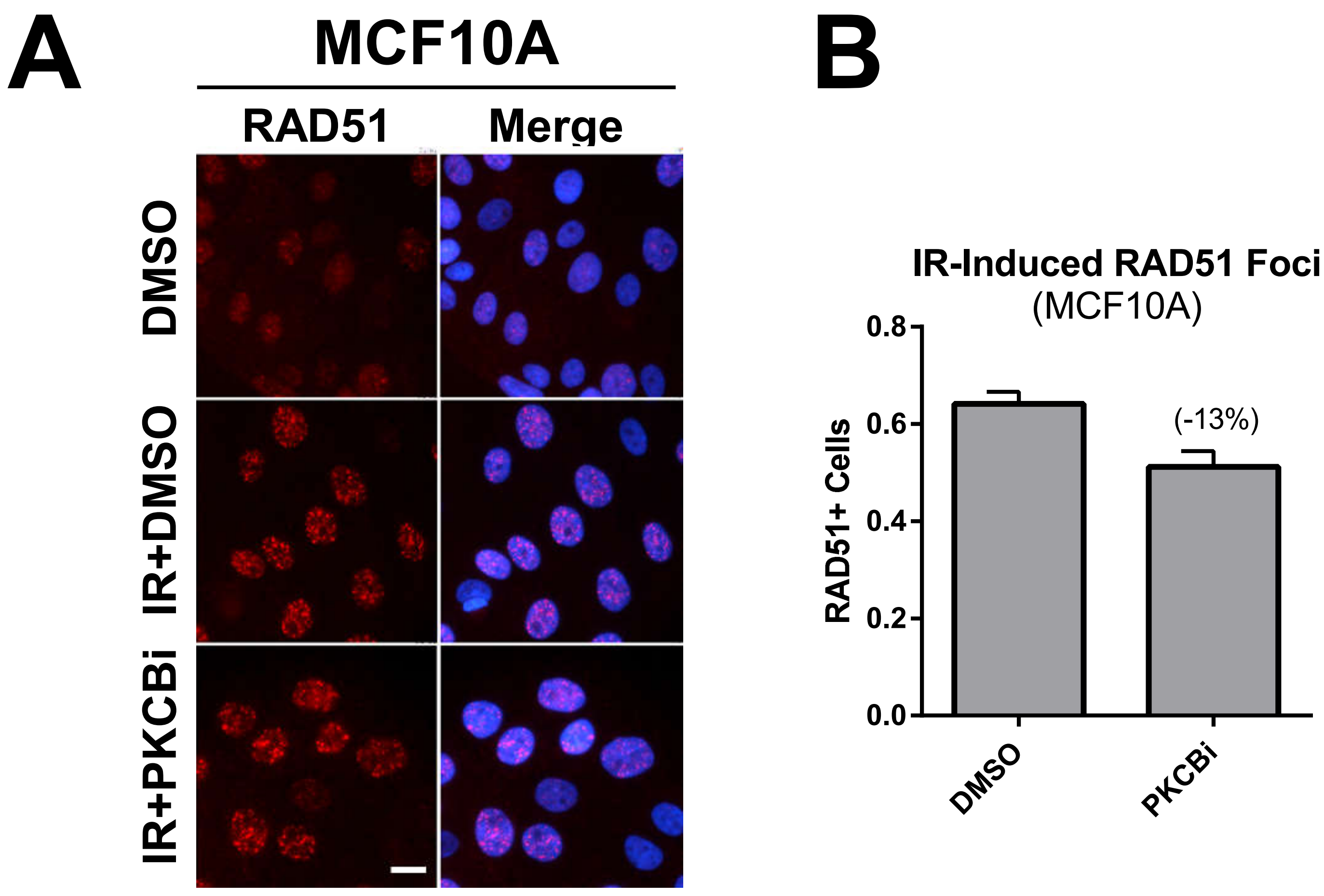

Figure S4. Minimal suppression of HR as indicated by RAD51 foci in non-malignant MCF10A cells.

A Images of radiation-induced Rad51 foci formation. Cells were pre-treated with 5 μM LY317615 for 4 hours, irradiated at 5 Gy, and then allowed to recover for 4 hours before immunostaining for Rad51 (red) and nuclei (blue). Scale bar = 10 μm.

B Quantification of images in A, when considering that cells with more than 10 Rad51 foci are deemed as being positive.
